# Effect of a Snack Bar Optimized to Reduce Alcohol Bioavailability: A Randomized Controlled Clinical Trial in Healthy Individuals

**DOI:** 10.1089/jmf.2019.0228

**Published:** 2020-04-08

**Authors:** Joseph M. Fisher, Thomas M.S. Wolever, Janice E. Campbell, Adish Ezatagha, Jarvis C. Noronha, Alexandra L. Jenkins

**Affiliations:** ^1^ Zeno Functional Foods, LLC, Redwood City, California, USA.; ^2^ INQUIS Clinical Research, Ltd., Toronto, Ontario, Canada.

**Keywords:** BAC, binge drinking, first pass metabolism, food, gastric emptying, intoxication, protein

## Abstract

Alcohol intoxication impairs judgment and reaction times and the level of blood alcohol concentration (BAC) is highly correlated with accidents and injury. We hypothesized that a food optimized to delay gastric emptying, a reduced alcohol bioavailability bar (RABB), would decrease postprandial BAC and alcohol bioavailability with greater caloric-efficiency than control foods. Therefore, we evaluated the RABB in a randomized, crossover trial in 21 overnight fasted healthy adults (10 male, 11 female). Just before consuming a moderate dose of alcohol (0.3–0.35 g/kg body weight), participants ate either (1) no food (NF, 0 kcal), (2) the RABB (210 kcal), (3) a savory snack mix (SSM, 210 kcal), or (4) a multicomponent meal (MCM, 635 kcal) and their BAC was measured over 90 minutes using a breathalyzer, the primary endpoint being peak BAC (pBAC). pBACs were analyzed by repeated measures analysis of variance (ANOVA) (*F* = 107.5, *P* < .0001) with the differences between means assessed using Tukey's honestly significant difference test. The pBAC of each group was different (*P* < .001) from all other groups (NF = 0.064 ± 0.003, SSM = 0.047 ± 0.002, RABB = 0.031 ± 0.002, MCM = 0.020 ± 0.002%; mean ± standard error of the mean). Furthermore, the bioavailability of alcohol over 90 minutes (BA90) was reduced compared to the NF group by similar margins (SSM = 22.0 ± 2.2, RABB = 45.0 ± 3.8, MCM = 67.9 ± 3.1%) with the mean BA90 of each group different from all other groups (*P* < .001). Compared to the NF condition, the average reduction of pBAC per 100 calories of food consumed was higher for the RABB (24.0%) than either the SSM (11.8%) or the MCM (10.7%). This study demonstrates that the RABB can reduce both pBAC and alcohol bioavailability with high caloric-efficiency.

## Introduction

The use of alcohol is an important risk factor for disease burden and mortality around the world through both its chronic and acute effects.^1–4^ Chronic alcohol use is associated with the morbidity of liver and cardiovascular disease, many types of cancer, infectious, and other diseases.^5–11^ Acute alcohol intoxication causes impaired judgment and coordination, slowed reaction times, and the level of blood alcohol concentration (BAC) is highly correlated with accidents and associated injury.^12^ Although there has been some evidence that low to moderate alcohol consumption can be beneficial in reducing the severity of both cardiovascular disease and diabetes, recent evidence suggests that the safest level of alcohol consumption is nil.^1,13,14^

The economic costs attributable to alcohol consumption amount to more than 1% of the gross national product in middle- to high-income countries, largely due to the economics of the social harm caused.^15^ Due to these negative consequences, governmental agencies around the world have implemented many policies and initiatives to curtail alcohol use, including age restrictions, driving limits, taxation, as well as prevention programs targeted to minors, schools, workplaces, families, and communities; these have all had limited success.^16^

An individual's BAC is the result of many factors, including their body composition, physiology, the quantity and timing of the alcohol consumed, and the alcohol concentration in their drink.^17^ Furthermore, food consumption before or with an alcoholic drink influences both the speed and amount of alcohol absorption by reducing the rate of gastric emptying and enhancing gastric and hepatic first pass metabolisms.^18,19^ Eating a ∼700 kcal meal just before alcohol consumption was shown to reduce peak BAC (pBAC) and total bioavailability by over 50%.^20^ However, consuming these many calories before drinking may not be realistic in typical alcohol use scenarios. Furthermore, there is scant information available regarding the effects of different foods, or macronutrients, on alcohol absorption and it is currently considered that the primary determinant is the total number of calories consumed.^20–22^

The rate of gastric emptying is influenced by the volume and physical form (liquid vs. solid) of food consumed as well as its macronutrient composition.^23^ Milk proteins, insoluble fibers, and polysaccharides that induce viscosity have all been shown to have pronounced effects on gastric emptying.^24–28^ Since the gastric emptying rate appears to significantly influence alcohol absorption, and may do so primarily through enhancing gastric first pass metabolism, the consumption of foods that slow gastric emptying may be advantageous at modulating alcohol pharmacokinetics.^18,19,29^ In this study, we report the formulation and evaluation of a food bar enriched in milk protein and insoluble fiber (reduced alcohol bioavailability bar, RABB) on BAC compared to both an isocaloric control as well as a hypercaloric multicomponent meal (MCM). We hypothesized that the RABB would reduce the bioavailability of alcohol with higher caloric-efficiency than the control test meals.

## Materials and Methods

### Study design and protocol

The study was designed as a four-way crossover, randomized, controlled trial carried out at a single site by an independent clinical research organization (INQUIS Clinical Research, Ltd., Toronto, Ontario, Canada). Due to the nature of the study, it was open-label and the subjects were aware of the types of food they were consuming although they were not informed as to the functional purpose of the RABB or the underlying study goals; the order of the food intervention arms was randomized using an online program (https://www.randomizer.org). The study interventions took place between March 12 and June 11, 2019. Subjects reported to the test site once for consent and screening, and then for four more visits no <5 days apart.

The study was conducted, and informed consent obtained, in compliance with all pertinent clinical and regulatory guidelines, including the Declaration of Helsinki, and was reviewed and approved by an external Institutional Review Board (Advarra IRB, Aurora, Ontario, Canada). Before recruitment of the first subject, the trial was registered on https://clinicaltrials.gov (identifier: NCT03867812).

The study protocol consisted of four visits to the clinic, each after an overnight fast. The night before a test, the subject consumed a standardized dinner consisting only of food provided to them before each visit. They were instructed to have an overnight water-only fast and to come to the clinic on an empty stomach; any deviations to this procedure were to be reported. For the test, the subject ate a test food(s), or no food (NF), along with an 8 oz glass of water over 10 minutes. Just after consuming the test food, the subject rated the palatability of the meal on a visual analog scale from (0) “unpalatable” to (100) “very palatable.” Five minutes after consuming the study interventions, the subject drank a 20% alcohol by volume cocktail consisting of 80-proof vodka and noncaloric tonic water over 10 minutes.

The size of the cocktail was adjusted according to the sex and bodyweight of the subject; alcohol dosage was 0.3 g (females) and 0.35 g (males) per kg of bodyweight. The amount of alcohol consumed was approximately equivalent to two standard drinks for a 75 kg man (about 3 oz of 80-proof spirit). After the cocktail was consumed, the subject rinsed their mouth and throat with water twice and BAC measurements were then taken every 10 minutes thereafter until 90 minutes from the start of the drink had elapsed, or until a clear pBAC was established. BAC determinations were estimated using a calibrated law enforcement grade breathalyzer (Alco-Sensor IV; Intoximeters) with measurements repeated at each timepoint two (if both within 0.002% BAC) or three times. After completion of the BAC time-course, subjects were provided with food, water, and transportation (if desired) and kept onsite until their BAC was <0.04%. Adverse events related to the test procedure were recorded as were any specific causes if determinable.

### Test subjects

Subjects with a history of moderate alcohol usage were identified and recruited from the local area around Toronto, Ontario, Canada from February to June 2019. Participants were enrolled from those who met the following inclusion criteria: (1) male or female, (2) aged 25–64, (3) body mass index 20–30 kg/m^2^, (4) blood pressure <140 mmHg (systolic) and <95 mmHg (diastolic), (5) social drinkers with an average of two or fewer drinks (1.5 oz 80-proof equivalents define one drink) per day, and (6) ability and willingness to comply with the protocol, including dietary, activity, or other restrictions throughout the duration of the trial.

Key exclusion criteria were as follows: (1) a history of alcohol abuse or having one or more episodes of “binge drinking” over the previous 30 days (“binge drinking” as defined by the US National Institute on Alcohol Abuse and Alcoholism), (2) history of cardiovascular, metabolic, respiratory, renal, gastrointestinal, or hepatic disease, (3) presence of any health conditions that would prevent fulfillment of the study requirements, (4) for women, being pregnant, lactating, or testing positive using a urine pregnancy test before or during the trial, (5) East Asian descent and/or a history of a flushing reaction when consuming alcohol, (6) smoker, (7) use of antibiotics within 4 weeks of the study start, (8) a major trauma or surgical event within 3 months of screening, (9) history of cancer within the past 2 years, except for nonmelanoma skin cancer, (10) history of mental illness, seizures, or the use of psychoactive medications, or other medications, which may affect the trial results, and (11) intolerance, sensitivity, or allergy to any of the study food products.

**FIG. 1.**
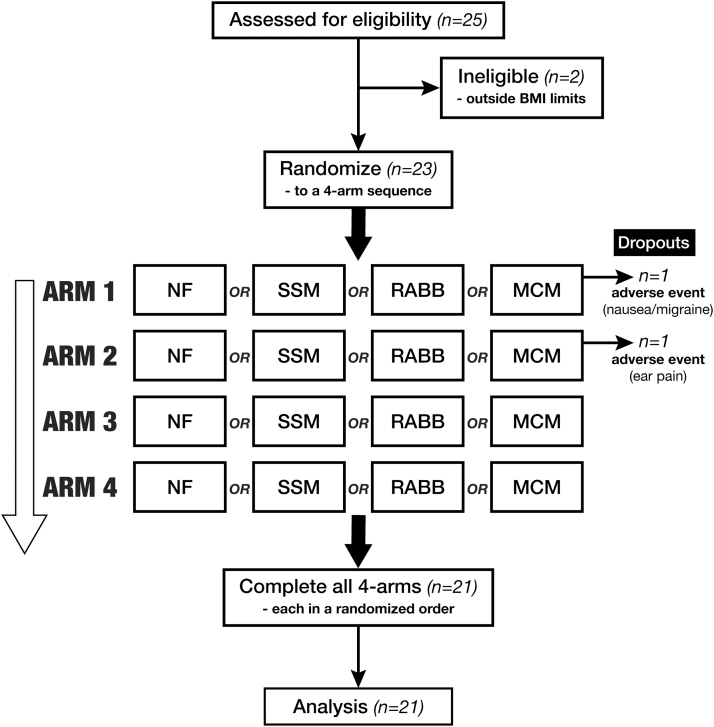
Flowchart of the study procedures. Each subject visited the clinic on four occasions, for each arm of the study, with a >5-day washout period between visits.

In total, 25 subjects were screened, of which 23 were enrolled; 2 subjects dropped out due to adverse events leaving 21 subjects (10 male, 11 female) completing all four experimental arms. The summary of the trial flow is seen in the consort diagram (Fig. 1) with the subject characteristics listed in Table 1.

**Table 1.** Basic Characteristics of the Trial Subjects

SN	Sex	Ethnicity	Age (years)	Weight (kg)	BMI (kg/m^2^)	SBP (mmHg)	DBP (mmHg)
1	Female	Caucasian	25	54.3	20.2	113	70
2	Female	Caucasian	26	62.1	23.8	113	84
3	Female	South Asian	26	65.2	24.7	116	75
4	Female	Caucasian	28	60.2	24.4	106	72
5	Female	Latin American	29	67.7	24.2	110	72
6	Female	Caucasian	37	65.6	22.7	113	75
7	Female	Caucasian	42	68.2	23.6	100	65
8	Female	Caucasian	51	65.5	27.0	101	60
9	Female	Black	52	66.4	23.3	128	93
10	Female	Caucasian	64	58.4	24.8	113	65
11	Female	Caucasian	64	53.8	21.4	121	58
12	Male	Caucasian	25	69.2	20.8	123	63
13	Male	Caucasian	27	88.2	27.1	135	75
14	Male	Caucasian	28	88.1	26.0	134	62
15	Male	Caucasian	30	77.1	26.7	137	71
16	Male	Latin American	31	72.5	26.5	122	60
17	Male	Caucasian	34	74.9	24.0	130	69
18	Male	Latin American	36	80.3	23.9	120	68
19	Male	South Asian	40	63.8	20.8	130	83
20	Male	Caucasian	48	88.7	29.2	124	79
21	Male	Caucasian	50	74.5	27.0	120	75
Mean (SD)			37.8 (12.5)	69.7 (10.3)	24.4 (2.4)	119.5 (10.6)	71.1 (8.9)

BMI, body mass index; DBP, diastolic blood pressure; Ethnicity, race or nationality; SBP, systolic blood pressure; SD, standard deviation; SN, subject number.

### Foods

All foods used in the study were either purchased from local (Toronto, Ontario, Canada) supermarkets or formulated in a test kitchen (Zeno Functional Foods, LLC, Redwood City, California, USA). The pretest standardized dinner was 800–940 calories and consisted of vegetable lasagna (Amy's), tomato cup-a-soup (Lipton), fruit cup (Del Monte), potato chips (Lays), chocolate pudding (Conagra), and an optional soft drink (Coca-Cola). The test foods included a savory snack mix (SSM, honey nut Chexmix [General Mills], a MCM (5 cheese Bistro Crustini [Stouffer's], strawberry yogurt [Oikos], orange juice [Tropicana], and an oatmeal cookie [Dad's]), and the RABB. The RABB was formulated using industry grade food ingredients, each accompanied with a certificate of analysis, and the final formulated bars were tested for water activity, the presence of mold and pathogens, and then stored at 4°C until use at the trial site.

The RABB consisted of milk protein hydrolysate, almonds, white chocolate (cane sugar, cocoa butter, milk powder, soy lecithin, and vanilla extract), protein crisps (whey protein, rice starch, and calcium carbonate), allulose syrup, tagatose, insoluble oat fiber, water, humectant (grape juice concentrate, and rice starch), acacia gum, natural flavors, and sea salt. The macronutrient compositions of the test foods are listed in Table 2.

**Table 2.** Nutritional Characteristics of the Test Foods

Food	Weight (g)	Energy (kcal)	Protein (g)	Fat (g)	CHO (g)	Fiber (g)
SSM	48.5	210	3.2	5.7	37.2	0.8
RABB	70.0	210	20.0	9	30.0	5.0
MCM	496.8	635	22.0	20.5	92.0	1.5

CHO, total carbohydrates; Fiber, insoluble fiber; MCM, multicomponent meal; RABB, reduced alcohol bioavailability bar; SSM, savory snack mix.

### Statistical analysis

The analytic and statistical analysis software used was Microsoft Excel which included the Real Statistics resource pack add-in. Significance testing was two-tailed and included repeated measures analysis of variance (ANOVA) with Tukey's *post hoc* testing, pairwise *t*-tests, and other appropriate tests along with graphical analysis. For pBAC as well as bioavailability of alcohol over 90 minutes (BA90) analysis, both aggregation by timepoint, as well as intraindividual calculations (when comparing peak reductions of pBAC compared to the NF condition) were used. The BA90 for a time-course was estimated using the trapezoid methodology to calculate the incremental area under the curve. Data were aggregated and analyzed independently by both J.F. and A.L.J. and any discrepancies were reconciled. Microsoft Excel and Adobe Illustrator software were used for plotting and figure preparation.

## Results

### Subject characteristics and trial sequence

Twenty-three subjects were randomized into the study, of which 21 completed all 4 experimental arms (Fig. 1) (10 men and 11 women, age 37.8 ± 12.5 [mean ± standard deviation], range 25–64). Two participants dropped out due to adverse events (nausea and ear infection) after completing the first or second arm, respectively. In addition to those who dropped out, two other participants experienced minor adverse events (including headache, allergic reaction, and a sore throat), but completed the experimental procedures. The nausea and headache may have been related to the study procedure and the ingestion of alcohol, but none of the adverse events was deemed to be related to the consumption of the interventional product.

### Test foods and RABB

The foods selected for testing as comparators to the RABB were representative of those typically consumed before or during the consumption of alcohol in western societies, a SSM and a moderately sized MCM similar to those used in comparable studies.^20^ To select a suitable RABB candidate, a series of bar formulations were developed that targeted total energy content of 200–250 calories, 20 g of total protein (>75% milk derived), high (>4 g) insoluble fiber content (primarily from oat hulls), with favorable organoleptic characteristics, using standard food industry ingredients and methodologies.^30^ The RABB selected had a much higher level of both protein enrichment per calorie and insoluble fiber content than did the other foods used in testing; the nutritional characteristics of all the foods are listed in Table 2.

Foods tested in the trial were rated as highly palatable by the participants using a 0–100 visual-analog scale (SSM, 68 ± 5, RABB, 66 ± 5, MCM, 78 ± 3, means ± standard error of the mean [SEM]). Although the average rating for the MCM was higher than that for the SSM or RABB, the difference did not achieve statistical significance as determined by ANOVA (*F* = 2.01, *P* = .14) or by pairwise *t*-tests (all *P* > .05).

### BAC time-profiles

**FIG. 2.**
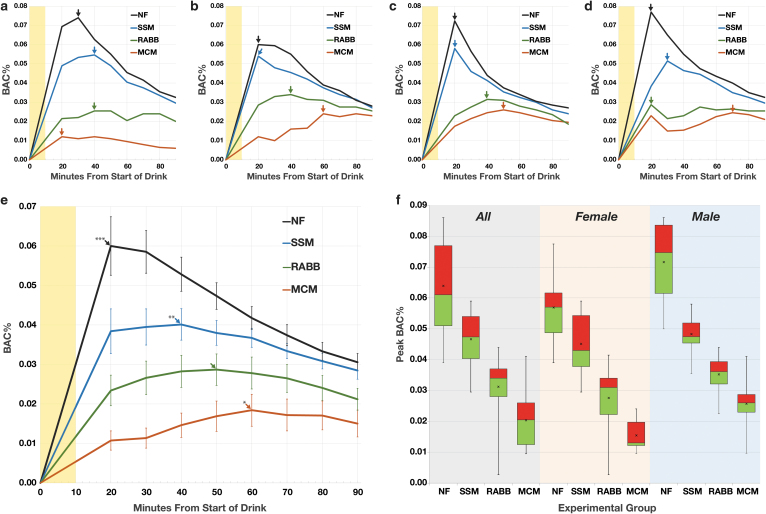
BAC measurements of the participants. **(a–d)** Example BAC-time plots of four participants, 10 minute duration of drinking is shaded *yellow*, pBAC for each plot indicated by *arrow*. **(a)** Sixty-four-year-old female. **(b)** Forty-two-year-old female. **(c)** Forty-year-old male. **(d)** Thirty-one-year-old male. **(e)** Graphs of the aggregated BAC-time plots for all 21 participants. Each datapoint represents the mean of all BAC values at a given timepoint, error bars are the 95% confidence interval. *Arrows* indicate the peak of the aggregated BACs, significant difference from RABB indicated by *P < .01, **P < .001, ***P < .0001. **(f)** Boxplots of the pBACs for all participants (*n* = 21), females (*n* = 11), and males (*n* = 10). The *line* separating the *red* and *green* is the median, the “x” marks the mean, *box top* and *bottom* marks upper and lower quartiles, whiskers define the total range of values. BAC, blood alcohol concentration; pBAC, peak BAC; RABB, reduced alcohol bioavailability bar.

Each subject demonstrated a unique BAC-time profile for each test condition. Typical BAC-time plots from four subjects illustrating the heterogeneity of responses are shown in Figure 2a–d; these were consistent with those seen in similar studies.^20^ Two key variables in these plots were the pBAC and the time to pBAC (TTP, Fig. 2a–d, arrows), which varied between subjects. For all conditions, the range of pBACs (0.003–0.086%) and TTPs (20–100 minutes) observed was considerable. When the BAC-time profiles of all subjects are aggregated by timepoint (Fig. 2e), two clear patterns emerged. The first was that the peaks of the time-aggregated groups (tapBAC) followed the same pattern common to most individuals with (mean ± SEM) NF (0.060 ± 0.004) > SSM (0.040 ± 0.002) > RABB (0.029 ± 0.002) > MCM (0.018 ± 0.002%). The second was that the time to reach the tapBAC increased as the tapBAC decreased (Fig. 2e, arrows), increasing from NF (20 minutes) to SSM (40 minutes) to RABB (50 minutes) to MCM (60 minutes). When looking at the distribution of the individual pBACs, regardless of what the value of their TTPs (Fig. 2f), the same pattern is evident for all subjects or when broken out into female and male subgroups. Of note, the mean pBACs for the NF condition were significantly (*P* = .017) lower for the females (0.057%) than the males (0.072%); this was also seen for the MCM condition (*P* < .01) with female mean pBACs (0.015%) less than males (0.026%). Between sex, pBAC means for the SSM and RABB groups were not significantly different (*P* > .05).

### Food effects on pBAC, TTP, and alcohol bioavailability

The effects of consuming the RABB, its isocaloric control (SSM), and a larger meal (MCM) before alcohol dosing are summarized in Table 3. The mean pBAC of each group was different from all others with a high degree of significance (repeated measures ANOVA, *F* = 107.5, *P* < .0001, Tukey's test each *P* < .05). In terms of the reduction of pBAC compared to the fasting (NF) condition (pBACr%), the mean of each individual's % reduction for the RABB was over 50% and closer to the MCM than to the isocaloric control. Furthermore, the range of the pBACr% of the RABB more closely paralleled the MCM; the SSM group range had a negative value as one subject's pBAC for the SSM condition was greater than that of NF. The pBACr% for each 100 calories of food consumed (pBACr%Kcal) was about the same for the SSM (11.8%) and MCM (10.7%), while the RABB was over two times as great (24.0%).

**Table 3.** Food Effects on Peak Blood Alcohol Concentration and Bioavailability

Food	Kcal	pBAC	pBACr%	pBACr% range	pBACr%Kcal	TTP	BA90	BA90r%
NF	0	0.064 (0.003)^*^	NA	NA	NA	27.6 (1.9)^***^	3.77 (0.14)^*^	NA
SSM	210	0.047 (0.002)^*^	24.7	−18.0–57.4	11.8	40.0 (3.9)^†^	2.91 (0.10)^*^	22.0
RABB	210	0.031 (0.002)	50.4	22.1–93.2	24.0	42.9 (3.4)	2.08 (0.14)	45.0
MCM	635	0.020 (0.002)^*^	67.7	39.2–88.7	10.7	62.4 (4.6)^**^	1.19 (0.12)^*^	67.9

Results reported as mean or mean (standard error of the mean), *n* = 21. All statistical differences are two-tailed Tukey's test comparisons to the RABB: ^*^P < .0001, ^**^P < .001, ^***^P < .01, ^†^*P* > .05 (not significant).

BA90, the average bioavailability of alcohol over 90 minutes as measured by the area under the BAC-time curve; BA90r%, the average % reduction of the BA90 compared to the NF condition; BAC, blood alcohol concentration; Food, experimental arm; Kcal, energy in kilocalories; NA, not applicable; NF, no food; pBAC, mean peak BAC%; pBACr%, mean % reduction of the pBAC compared to the NF condition; pBACr%Kcal, the average pBACr% per 100 calories of food; pBACr% range, the range of pBACr% values; TTP, the average time to pBAC from the start of drinking.

There was considerable variability among the subjects in terms of the time to achieve their pBACs, but the mean TTPs of the groups showed a clear pattern with MCM > RABB > SSM > NF. Although the mean TTPs of the NF and MCM groups were statistically different from all other groups (*P* < .05), the difference between the RABB and SSM groups was not significant (*P* = .55). The bioavailability of the alcohol dose over 90 minutes (BA90) was estimated by determining the area under the BAC-time profile for each subject. The mean BA90s for the groups followed the same pattern as that of the pBACs, with NF > SSM > RABB > MCM, each mean being significantly different from the others (repeated measures ANOVA, *F* = 129.2, *P* < .0001, all Tukey's test *P* < .001). The mean percent reduction of bioavailability compared to the NF condition (BA90r%) also was in line with the pBAC results, with the RABB's reduction (45%) over two times as much as the isocaloric SSM (22%).

## Discussion

The most common approaches to limiting alcohol's negative consequences have been through educational and regulatory efforts to both minimize total intake as well as to limit the pBACs of those consuming it. In particular, minimizing pBAC is of great consequence as it is highly correlated with the frequency of multiple types of accidents, their associated harm, and violence.^12,31^ Although consuming food before and during drinking is a well-established method of limiting pBAC, there has to date been relatively little research and development efforts focused on creating foods optimized for this purpose. This study demonstrates the feasibility of creating such a food, the RABB, and quantifies its effects on alcohol pharmacokinetics in a group of healthy men and women.

The formulation for the RABB used in this study was based on an extensive review of the scientific literature regarding the relationship between gastric emptying, gastric and hepatic first pass alcohol metabolism, and food components that may be influential in this regard. Based upon these learnings, in conjunction with preliminary clinical case study data using prototype RABBs, a final test food was selected.^32^ It was very important that the RABB selected was “snack-size,” with <250 calories, as it is important to many individuals consuming alcohol to limit their caloric intake. In many instances, individuals attempt to minimize the food calories consumed before drinking, out of concern about potential weight gain, which has given rise to a specific behavioral pattern termed “drunkorexia.”^33^

Similar to the findings of other studies involving food and alcohol, the results here demonstrated that any food consumption before drinking reduces both pBAC and alcohol bioavailability; the order of mean pBACs showed a clear pattern where NF > SSM > RABB > MCM, each significantly (*P* < .05) different from each other. The mean TTPs had the opposite pattern, with MCM > RABB > SSM > NF; however, the difference between the RABB and SSM was not significant (*P* > .05). This observation highlights the variability of time (range: 20–100 minutes) to achieve the pBAC under the various experimental conditions and illustrates how the TTP is not perfectly correlated with the magnitude of the pBAC for an individual.

Of note, the average pBACs of the females, for the NF and MCM conditions, were significantly lower than the males. For the NF condition, this 20.6% decrease was unexpected as women have demonstrated greater pBAC sensitivity than men to a moderate dose of alcohol in comparable studies^34^ and their dosage here was reduced accordingly (0.35–0.30 g/kg, a 14.3% reduction). It appears that the female and male participants in this study responded more similarly to each other which might be attributable to sampling issues in this small sized trial. Regardless of this difference, the effect of the RABB relative to the NF condition was comparable between the sexes with mean reductions of pBACs (females = 51.5, males = 50.8%) almost identical in size. Finally, the total bioavailability of the alcohol dose over 90 minutes was reduced by the RABB with much greater caloric-efficiency than the other foods tested; for each 100 calories consumed, the BA90 was diminished by 10.5% (MCM), 10.7% (SSM), and 21.4% (RABB). This study strongly supports the notion that when it relates to effects on alcohol absorption, all foods are not the same.

In conclusion, the RABB represents the first example of a food specifically designed to reduce alcohol absorption. For the healthy adults in this study, the consumption of the RABB before having a moderate dose of alcohol resulted in a near 50% reduction in both pBAC and bioavailability. Eating a RABB before drinking is an easy to implement, and calorically-efficient, adjunctive method to limit the effects of alcohol when this is desirable.

## Authors' Contributions

J.M.F., A.L.J., and T.M.S.W. were responsible for study concept and trial design. J.E.C., A.E., and J.C.N. were responsible for carrying out the trial and data handling/validation. Both A.L.J. and T.W. as well as J.M.F. independently analyzed and interpreted the data. J.M.F. drafted the article and all parties reviewed and critically revised it.

## Ethical Approval

This study was designed and conducted in accordance with the ethical standards as put forth in the Declaration of Helsinki 1975, as revised in 2008. Since the trial protocol involved the administration of alcohol, the study design also conformed to the recommendations from the US National Institute on Alcohol Abuse and Alcoholism (NIAAA).^35^ The study protocol (GIL-1842), informed consent forms, and associated documents were reviewed and approved by a third-party Institutional Review Board under Health Canada purview (Advarra IRB [approved February 27, 2019, Pro00032144]).
